# Epidemiological and Clinical Characteristics Associated with COVID-19 Severity Among Hospitalized Patients in the United Arab Emirates: A Retrospective Multicentre Study

**DOI:** 10.1007/s44197-024-00206-8

**Published:** 2024-02-26

**Authors:** Najlaa Al-Bluwi, Razan Agha, Ankita Shukla, Rouba Karen Zeidan, Hamzah AlZubaidi, Manal Awad, Amal Hussein, Muzan Abdelbagi, Khaled AlSayed, Mohamad B. Alebaji, Mahasin Shaheen, Laila Salameh, Bassam Mahboub, Hady Elkhodary, Riyad Bendardaf, Ghada Mohammed, Dima Wardat, Zahraa Al-Hano, Hajir I. Amara, Mohamed Saleh Alhajjaj, Qutayba Hamid, Rabih Halwani, Basema Saddik

**Affiliations:** 1 Sharjah Institute of Medical Research, University of Sharjah, Sharjah, United Arab Emirates; 2 College of Pharmacy, University of Sharjah, Sharjah, United Arab Emirates; 3 Department of Orthodontics, Pediatric and Community Dentistry, College of Dental Medicine, University of Sharjah, P.O. Box No 27272, Sharjah, United Arab Emirates; 4 Department of Family and Community Medicine and Behavioral Sciences, College of Medicine, University of Sharjah, Sharjah, United Arab Emirates; 5 Department of Clinical Sciences, College of Medicine, University of Sharjah, Sharjah, United Arab Emirates; 6 Al Qassimi Hospital, Sharjah, United Arab Emirates; 7 Rashid Hospital, Dubai Health Authority, Dubai, United Arab Emirates; 8 American Hospital Dubai, Dubai, United Arab Emirates; 9 University Hospital Sharjah, Sharjah, United Arab Emirates; 10 Epidemiology Unit, University of Sharjah, Sharjah, United Arab Emirates; 11 School of Population Health, University of NSW, Sydney, Australia; 12 School of Medicine, Deakin Rural Health, Deakin University Faculty of Health, Warrnambool, Victoria, Australia

**Keywords:** COVID-19, Severity, Epidemiological, Clinical, Predictor

## Abstract

**Objectives:**

To investigate the clinical and epidemiological factors associated with severe COVID-19 cases in hospitalized patients across two emirates within the United Arab Emirates (UAE).

**Methods:**

A retrospective observational analytical study analysed data from 738 medical records and conducted 573 in-depth interviews with patients hospitalized across multiple healthcare centers in the UAE, between 29 January 2020 and 14 October 2021. Regression analysis predicted risk factors for COVID-19 severity.

**Results:**

Main risk factors identified were crowding (aOR 1.919; 95%CI 1.144, 3.221), obesity (aOR 2.383; 95%CI 1.332, 4.263), diabetes (aOR 11.14; 95%CI 2.653–46.797), severe dehydration (aOR 3.219; 95%CI 2.161, 4.795), cough or sore throat (aOR 1.607; 95%CI 1.032, 2.502), shortness of breath (aOR 1.921; 95%CI 1.294, 2.853), increased days from symptom onset to admission (aOR 1.055; 95%CI 1.006, 1.105), elevated ANC (aOR 1.263, 95%CI 1.121, 1.424), and AST/SGOT (aOR 1.055, 95% CI 1.016, 1.095). Protective factors included smoking (aOR 0.367; 95%CI 0.182, 0.740), first dose of COVID-19 vaccination (aOR 0.595; 95%CI 0.377, 0.93), higher oxygen saturation (aOR 0.853; 95%CI: 0.801, 0.907) and elevated ALC (aOR 0.540; 95%CI 0.323, 0.905).

**Conclusion:**

Identifying risk factors is crucial for high-risk individuals who may require closer monitoring to improve their outcomes. This can provide guidance for surveillance systems and early detection strategies to mitigate the impact of future outbreaks.

**Supplementary Information:**

The online version contains supplementary material available at 10.1007/s44197-024-00206-8.

## Introduction

On May 5, 2023, the World Health Organization (WHO) declared that the Coronavirus Disease 2019 (COVID-19) pandemic no longer posed a public health emergency of international concern [1]. However, the disease is still affecting populations worldwide, leading to over 680 million confirmed cases and 6.5 million deaths. As of June 20, 2023, the United Arab Emirates (UAE) had reported 1,067,030 confirmed COVID-19 cases and 2349 deaths [2].

COVID-19 severity and clinical manifestations vary across geographical locations and host factors. Severe patients may exhibit various symptoms and laboratory abnormalities including respiratory system symptoms, musculoskeletal problems, gastrointestinal complaints, mucocutaneous symptoms [3], lymphopenia, thrombocytopenia, abnormal liver function tests, and elevated inflammatory markers [4, 5].

Previous studies have identified predictors of COVID-19 severity including sociodemographic factors such as ethnicity, gender, and residence area [6, 7]; as well as comorbidities like diabetes, hypertension, kidney diseases, nervous system disorders, and obesity [8]. Smoking status has also shown conflicting associations with some studies suggesting it as a potential predictor of adverse COVID-19 outcomes [9], while others associate current smoking to a lower risk of severe COVID-19 [10]. The UAE's diverse population, influenced by variations in ethnicity, genetics, culture, and social practices may significantly impact the spread and severity of COVID-19 [7, 11].

Previous studies in the UAE focused primarily on COVID-19 severity using medical records [8] specific populations such as paediatric patients [12], were confined to a single emirate [8], or a single healthcare facility [13]. This study aims to address these gaps by using two methods of data collection across multiple healthcare centres in more than one emirate. This approach will obtain more precise and reliable data, explore novel relationships, increase the access and diversity to eligible study subjects, and improve generalization of the results.

## Methods

### Study Design and Setting

A retrospective observational study was conducted in four major hospitals in Sharjah and Dubai, UAE. These hospitals comprised a mix of private, semi-governmental, and governmental institutions offering a wide range of clinical services, and specialized ICU units. The private hospital offered 254 beds, while the semi-governmental hospitals provided 417 and 325 beds, respectively. The governmental hospital had 762 beds. These hospitals were selected for their capacity, range of specialties, and to ensure diverse representation in the study.

### Participants and Sample Size

This study included adults aged 18 years or older who tested positive for COVID-19 using the Real time Reverse Transcriptase Polymerase Chain Reaction (RT-PCR) test on nasal and/or pharyngeal swab specimens in laboratories, and who were admitted to one of the four selected hospitals between January 29, 2020, and October 14, 2021. Pregnant women and those unable to provide informed consent were excluded from the study. Each participating hospital provided a list of inpatients who met the inclusion criteria. Participants were then sorted in ascending order based on their hospital ID numbers. We performed a systematic random selection from these lists at predetermined regular intervals where every third person in the list was chosen.

For this study, a minimum of 385 patient records were needed, using the formula Sample Size = [z2 * p (1-p)] / e2, assuming a prevalence of 50%, a margin of error of 5%, and a confidence level of 95%. However, considering a non-response and incomplete response rate of 20%, the target minimum sample size was adjusted to 482 adults.

**Fig. 1:**
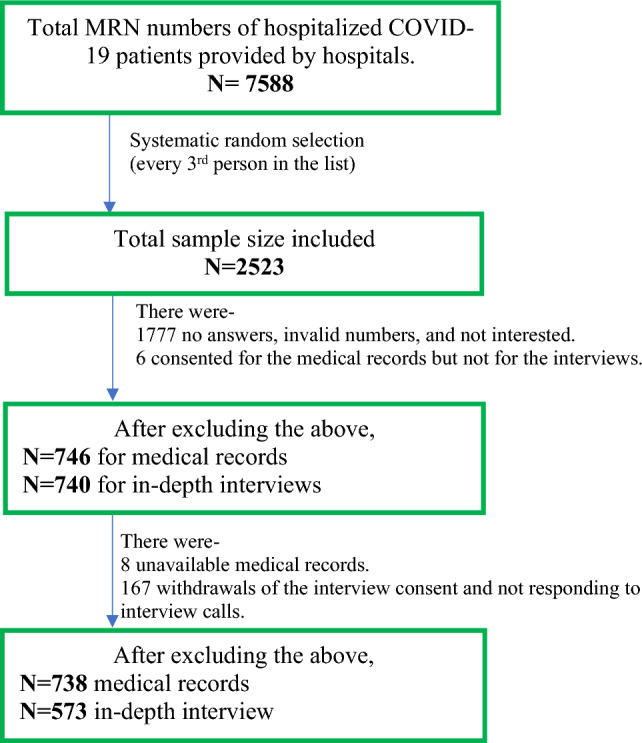
Description and breakdown of patients’ data

The final achieved sample was 738 for medical records and 573 for the detailed epidemiological telephone interviews. A detailed description of how the final sample size was achieved, as well as a breakdown of participants who were included, did not respond, did not consent, and the number of inaccessible medical records are presented in Fig. 1.

### Data Collection and Study Tools

The study utilized data from both medical records, as well as in-depth interviews with COVID-19 hospitalized patients. The interview guide was initially prepared in English and translated into Arabic and Urdu by the study team. Back-translations were performed to ensure equivalence reliability. Before being sent to five specialists for content validation, 15 adults piloted the interview guide. Following the pilot study, the research team modified the study guide slightly to enhance its efficacy and validity. To ensure inter-rater reliability all interviewers and data collectors underwent the same training sessions.

The process of data collection was performed as follows. First, patients or their next of kin were invited to participate through telephone calls. Those who expressed willingness and consent were offered a convenient interview date and time. Those who declined to participate in the study were recorded as non-response. Next, the participants' medical data were retrieved. Finally, the interviews were conducted on the agreed-upon day and time.

The structured interview which collected information on sociodemographic characteristics, behavioral risk factors, and vaccination status took approximately ten minutes to complete.

Medical data were extracted from each patient’s electronic health records using a standardized data collection form. This form was a modified version of the WHO/International Severe Acute Respiratory and Emerging Infection Consortium acute respiratory infections (ISARIC) case record form. It included the following sections: severity on admission, past medical history, BMI categories (underweight < 18.5 kg/m^2^, normal 18.5–24.9 kg/m^2^, overweight 25.0–29.9 kg/m^2^, and obese > 29.9 kg/m^2^) [14], radiological testing, symptoms, clinical measures, and laboratory test results. Any missing or uncertain records were clarified through direct communication with healthcare providers or participants.

COVID-19 severity on admission was categorized into four categories following the National Health Commission of China guidelines that were available when we designed the study. The classification was defined as follows: (i) Mild: presented with mild symptoms without radiographic features; (ii) Moderate: presented with fever, respiratory symptoms, and radiographic features; (iii) Severe: met one of the three criteria: (a) dyspnea, respiration rate (RR) greater than 30 times/min, (b) oxygen saturation less than 93% in ambient air, and (c) PaO2/FiO2 less than 300 mm Hg; and (iv) Critical: met one of the following criteria: (a) respiratory failure, (b) septic shock, or (c) multiple organ failure [15]. To simplify regression analysis, COVID-19 severity was further grouped into two categories: non-severe (including mild) and severe (including, moderate, severe, and critical).

### Data Analysis

Data were analysed using SPSS version 28 [16]. Descriptive data were analysed using counts and percentages, and normality was tested using the Kolmogorov–Smirnov test. Associations between categorical variables were explored using Chi-square and Fisher’s exact tests, while two group comparisons involving continuous variables were conducted using independent samples t-test and Mann–Whitney U tests. Multivariate binary logistic regression analysis was conducted to predict the odds of severe COVID-19. Independent variables included in the models were selected based on statistical significance, while variables with small-number observations were excluded. Four models were developed: the first focused on sociodemographic characteristics, smoking history and medical history; the second examined symptoms and clinical measures; the third analysed laboratory results; and the final model included all study variables.

## Results

In total, clinical data from 738 patient medical records were extracted. Out of these, detailed epidemiological telephone interviews were conducted with 573 patients. The larger number of nonresponses in the interviews was attributed to refusal to consent to interviews, withdrawals of the interview consent on the day of the interview, or failure to answer the call during the scheduled appointments. However, the incomplete responses in interviews were due to patients either not completing the entire interviews or refusing to answer specific questions. Additionally, the medical records had gaps due to variations in test performance and information recording among different hospitals.

### Sociodemographic Characteristics and Smoking History of Study Participants

Approximately half of the hospitalized COVID-19 patients were Arab (56.7%), employed (56.9%). and aged 30 to 60 years (61.5%). Most were married (79.1%) and had no family history of consanguinity (78.3%). While 76.5% identified themselves as non-smokers, 62.1% reported that no one in their family smoked (Table 1).

**Table 1:** Sociodemographic characteristics, smoking history, and past medical history of study participants

Variable (N)	n	%
Gender (737)		
Female	264	35.8
Male	473	64.2
Marital status (561)		
Single	55	9.8
Married	444	79.1
Divorced/widowed	62	11.1
Age in years (733)		
18–29	43	5.9
30–60	451	61.5
60 +	239	32.6
Age in years (median (IQR))	50.06 (23)	
Education level (560)		
Less than high school	193	34.5
High school	115	20.5
Bachelors	219	39.1
Postgraduate	33	5.9
Ethnicity (573)		
Asian	222	38.7
Arab	325	56.7
Others	26	4.5
Income/month AED (513)		
Less than 3000	99	19.3
3000–7999	137	26.7
8000–14999	102	19.9
15,000–29999	102	19.9
More than 30,000	73	14.2
Work status (552)		
Not working	238	43.1
Working	314	56.9
Occupation (314)		
Labour/cleaner/delivery	83	26.4
Admin work	84	26.8
Managerial	86	27.4
Business	37	11.8
Health care worker	24	7.6
Area of residence (559)		
Dubai	187	33.5
Sharjah/Ajman	354	63.3
Other emirates	18	3.2
Family history of consanguinity (557)	121	21.7
Crowding (542)		
1–2 person/room	374	69
3 or more person/room	168	31
Smoking status (565)		
Non-smoker	432	76.5
Former smoker	82	14.5
Current smoker	51	9
Smoking frequency (day/month) (132)		
Not regular (not every day)	50	37.9
Regular (all 30 days)	82	62.1
Smoking frequency (cigarettes/day) (105)		
1–5	54	51.4
6 and more	51	48.6
Anyone in the family smokes (563)	113	20.1
Past surgery (701)	144	20.5
BMI (618)		
Underweight	10	1.6
Normal	144	23.3
Overweight	226	36.6
Obese	238	38.5
Pre-existing medications (738)		
ACE inhibitors	59	8
ARBS	53	7.2
NSAID	53	7.2
Pressor support	10	1.4
Vaccination (573)		
Influenza	68	11.9
BCG	326	56.9
COVID-19 first dose	283	38.3
COVID-19 s dose	269	36.4
Previous measles infection	88	15.4
Radiological testing (738)		
Chest x-ray	665	90.1
CT	426	57.7
Other	22	3

### Past Medical History of Patients

**Fig. 2:**
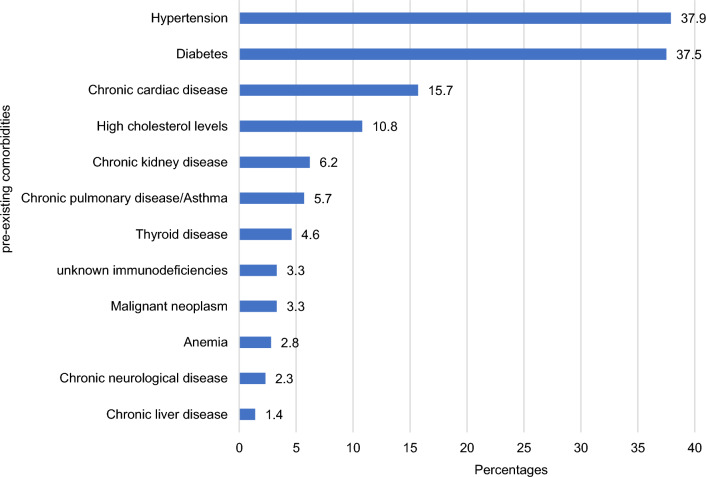
Percentages of pre-existing comorbidities among study participants (N = 738)

Hypertension (37.9%) and diabetes (37.5%) were the most common comorbidities among participants (Fig. 2). The majority were overweight or obese (75.1%). ACE inhibitors (8.0%) were the most frequently prescribed pre-existing medications, and 38.3% of patients had received the initial dose of the COVID-19 vaccine (Table 1).

### COVID-19 Severity Categories and Symptoms on Admission

**Fig. 3:**
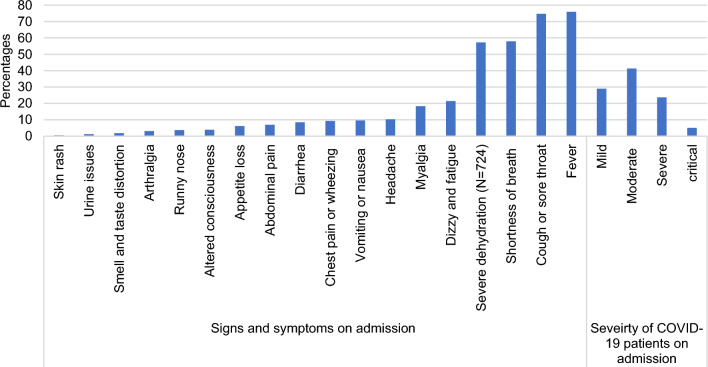
Percentages of COVID-19 severity and symptoms on admission of study participants (N = 738)

Around half of the hospitalized patients had a moderate form of COVID-19 (41.3%) on admission while 29% were classified as mild, 23.8% as severe and only 5% as critical. The most reported symptoms on admission were fever (75.9%), and cough or sore throat (74.7%) (Fig. 3). Lung abnormalities were diagnosed in 90.1% via chest X-ray and in 57.7% via CT scan (Table 1).

### Clinical Measures on Admission

Patients had a median duration of 4 days (IQR 5 days) from symptom onset to hospitalization. Most clinical measurements were within the normal range, except for a slightly elevated median systolic blood pressure at (128 mmHg) (IQR 25) and median body temperature of (37.4 °C) (IQR 1.4 °C) (Table 2).

**Table 2:** Clinical measures and laboratory results

Variable	N	Median^a^	IQR^a^	Normal range
Duration from symptoms to admission (days)	701	4	5	
Clinical measures on admission				
Systolic BP (mmHg)	723	128	25	< 120
Diastolic BP (mmHg)	721	77	15	< 80
Temperature (°C)	726	37.4	1.4	36.50–37.00
Heart rate HR (beats/min)	725	92	25	60–100
Respiratory rate RR (breaths/min)	720	20	6	12–20
Oxygen saturation SpO2 (%)	723	96	6	95–100
Glasgow coma score	716	15	0	15
Laboratory results				
Complete blood count CBC				
White blood count WBC (× 10^9^/l)	575	6.54	4.09	4.50–11.00
Hemoglobin Hb (g/dL)	576	13.10	2.40	12.00–15.00
Platelets (× 10^9^/l)	587	209.00	117.00	150.00–410.00
Absolute neutrophile count ANC (10^3/ul)	567	4.99	3.74	2.00–7.00
Absolute lymphocyte count ALC (10^3/ul)	580	1.00	0.67	1.00–3.00
Coagulation profile				
Prothrombin time PT (Secs)	361	12.30	2.30	9.70–11.80
Activated partial thromboplastin time aPTT (Secs)	319	36.32^b^	± 6.11^b^	25.10–37.70
International normalized ratio INR	358	1.03	0.15	< 1.10
D-dimer (mg/l)	498	0.72	0.67	< 0.50
Electrolytes				
Sodium (mEq/L)	558	136.00	6.25	135.00–145.00
Potassium (mEq/L)	562	3.99	0.78	3.60–5.20
Renal function				
Creatinine (umol/L)	546	84.00	37.00	44.00–133.00
Urea (mmol/L)	514	5.80	4.38	1.70–8.30
Liver function				
Albumin (g/l)	519	31.00	11.00	34.00–52.00
Bilirubin, total (umol/l)	521	8.80	7.00	1.71–20.50
Alanine aminotransferase/serum glutamic-pyruvic transaminase ALT/SGPT (U/L)	525	37.00	36.50	14.00–41.00
Aspartate aminotransferase/serum glutamic-oxaloacetic transaminase AST/SGOT (U/L)	458	40.00	34.00	15.00–37.00
Triglycerides (mmol/L)	57	1.79^b^	± 0.67^b^	< 1.70
Gamma-glutamyl transferase GGT (U/L)	61	33.00	45.00	5.00–36.00
Inflammatory markers				
Erythrocyte sedimentation rate ESR (mm/1 h)	115	53.00	48.00	< 20.00
Ferritin (ng/mL)	485	368.00	553.00	26.00–300.00
Procalcitonin (ng/mL)	451	0.14	0.26	< 0.05
Lactate (mmol/L)	110	1.47	0.90	0.50–2.20
Lactate dehydrogenase LDH (U/L)	435	367.00	235.00	135.00–214.00
C-reactive protein CRP (mg/l)	548	74.00	104.50	0.10–3.00
Interleukins IL_6 (pg/mL)	27	24.40	87.50	0.00–36.00
Indicators of cardiac injury				
Troponin (ng/mL)	333	0.009	0.018	< 0.040
Creatinine kinase CK (U/L)	265	115.000	187.000	22.000–167.000
Immunoglobin G IgG (g/L)	0	0	0	6.00–16.00

^a^p value of Kolmogorov–Smirnov ≤ 0.05; data is summarized in median and interquartile rang

^b^p value of Kolmogorov–Smirnov > 0.05; data is summarized in mean and standard deviation

### Laboratory Results

Upon admission, coagulation testing profiles indicated prolonged prothrombin time and elevated d-dimer levels with median values of 12.3 s (IQR 2.3 s) and 0.72 mg/l (IQR 0.670 mg/L) respectively. Elevated median values of inflammatory markers were also observed, ESR at 53 mm/1 h (IQR 48 mm/1 h), LDH at 367 U/L (IQR 235 U/L), ferritin at 368 ng/mL (IQR-553), procalcitonin 0.14 ng/mL (IQR 0.26 ng/mL), and CRP 74 mg/L (IQR 104.5 mg/L). Other laboratory results, including renal function, liver function, electrolytes, indicators of cardiac injury, and complete blood count were within normal limits (Table 2).

### Bivariate Analysis

Men had a higher percentage of severe cases (74.1%) compared to women (65.0%). Divorced and widowed patients (75.8%) showed higher severity rates compared to married (68.0%) and single (52.7%) patients. Patients aged 65 years or older (74.2%) had a higher proportion of severe cases compared to those aged 30 to 59 years (72.1%) and 18 to 29 years (41.9%). Patients residing in other emirates (77.8%), or Sharjah/Ajman (74.4%) had higher severity rates compared to residents of Dubai (54.0%). Patients with a monthly income less than 15,000 AED (72.0%) had a higher proportion of severe COVID-19 than those with incomes ranging from 15,000 to 30,000 AED (58.4%) and more than 30,000 AED (69.9%). Living in residences with three or more people sharing one room (73.7%) was associated with higher severity rates compared to living in less crowded homes (65.1%). Non-smokers and former smokers (68.9%) had higher frequencies of moderate/severe cases compared to current smokers (59.8%) (Table 3).

**Table 3:** Sociodemographic characteristics, smoking history, past medical history, and symptoms associated with COVID-19 severity on admission

Variables	Non severe	Severe	P-value^a^
	N (%)	N (%)	
Gender			
Female	92 (35)	171 (65)	0.009
Male	121 (25.9)	347 (74.1)	
Marital status			
Single	26 (47.3)	29 (52.7)	0.024
Married	141 (32)	300 (68)	
Divorced/widowed	15 (24.2)	47 (75.8)	
Age in years			
18–29	25 (58.1)	18 (41.9)	< 0.001
30–60	125 (27.9)	323 (72.1)	
60 +	61 (25.8)	175 (74.2)	
Age (median (IQR))	46.12 (26)	51.58 (23)	< 0.001^b^
Education level			
≤ High school	98 (32.1)	207 (67.9)	0.234
Bachelors	67 (30.6)	152 (69.4)	
Postgraduate	15 (45.5)	18 (54.5)	
Ethnicity (570)			
Asian	67 (30.5)	153 (69.5)	0.727
Arab	109 (33.6)	215 (66.4)	
Others	8 (30.8)	18 (69.2)	
Income/month AED			
< 15,000	98 (28)	242 (72)	0.034
15,000–29999	42 (41.6)	59 (58.4)	
≥ 30,000	22 (30.1)	51 (69.9)	
Work status			
Not working	77 (32.6)	159 (67.4)	0.804
Working	99 (31.6)	214 (68.4)	
Occupation			
Labour/cleaner/delivery	24 (28.9)	59 (71.1)	0.62
Admin work	27 (32.1)	57 (67.9)	
Managerial	26 (30.6)	59 (69.4)	
Business	11 (29.7)	26 (70.3)	
Health care worker	11 (45.8)	13 (54.2)	
Family history of consanguinity	40 (33.1)	81 (66.9)	0.805
Area of residence			
Dubai	86 (46)	101 (54)	< 0.001
Sharjah/Ajman	90 (25.6)	261 (74.4)	
Other emirates	4 (22.2)	14 (77.8)	
Crowding			
1–2 person/room	130 (34.9)	242 (65.1)	0.048
3 or more person/room	44 (26.3)	123 (73.7)	
Smoking status			
Non-smoker/former smoker	159 (31.1)	352 (68.9)	0.021
Current smoker	24 (47.1)	27 (52.9)	
Smoking frequency			
Not regular (not every day)	17 (34.7)	32 (65.3)	0.527
Regular (all 30 days)	33 (40.2)	49 (59.8)	
Smoking frequency (cigarettes/day)			
1–5	19 (35.8)	34 (64.2)	0.577
6 and more	21 (41.2)	30 (58.8)	
Anyone in the family smokes	44 (38.9)	69 (61.1)	0.102
Past surgery	49 (34.0)	95 (66.0)	0.096
BMI			
Underweight	8 (80.0)	2 (20.0)	< 0.001
Normal	52 (36.4)	91 (63.6)	
Overweight	69 (30.7)	156 (69.3)	
Obese	53 (22.4)	184 (77.6)	
Comorbidities			
Chronic cardiac disease	31 (27.2)	83 (72.8)	0.602
Hypertension	65 (23.6)	211 (76.4)	0.009
Chronic pulmonary disease/asthma	11 (26.2)	31 (73.8)	0.655
Chronic kidney disease	15 (32.6)	31 (67.4)	0.603
Chronic liver disease	6 (60)	4 (40)	0.072^c^
Chronic neurological disease‡	4 (23.5)	13 (76.5)	0.789
Diabetes	67 (24.5)	206 (75.5)	0.031
Malignant neoplasm	9 (37.5)	15 (62.5)	0.365
Unknown immunodeficiencies	2 (8.3)	22 (91.7)	0.022
Anaemia	8 (38.1)	13 (61.9)	0.365
High cholesterol levels	23 (28.7)	57 (71.3)	0.92
Thyroid disease	9 (26.5)	25 (73.5)	0.717
Pre-existing medications			
ACE inhibitors	16 (27.1)	43 (72.9)	0.709
ARBS	14 (26.4)	39 (73.6)	0.639
NSAID	13 (24.5)	40 (75.5)	0.434
Pressor support	3 (30.0)	7 (70.0)	0.957^c^
Vaccination			
Influenza	28 (41.2)	40 (58.8)	0.095
BCG	121 (37.3)	203 (62.7)	0.003
COVID-19 first dose	106 (37.7)	175 (62.3)	< 0.001
COVID-19 s dose	101 (37.8)	166 (62.2)	< 0.001
Previous measles infection	26 (29.5)	62 (70.5)	0.551
Symptoms on admission			
Fever	132 (23.7)	424 (76.3)	< 0.001
Cough or sore throat	138 (25.3)	408 (74.7)	< 0.001
Shortness of breath	74 (17.5)	348 (82.5)	< 0.001
Severe dehydration	62 (15.1)	348 (84.9)	< 0.001
Dizzy and fatigue	48 (30.4)	110 (69.6)	0.721
Myalgia	37 (27.4)	98 (72.6)	0.605
Headache	26 (34.2)	505 (65.8)	0.314
Vomiting or nausea	30 (42.3)	41 (57.7)	0.011
Chest pain/chest wheezing	13 (19.7)	53 (80.3)	0.074
Diarrhoea	23 (37.1)	39 (62.9)	0.155
Abdominal pain	21 (42.0)	29 (58.0)	0.04
Appetite loss	16 (35.6)	29 (64.4)	0.336
Altered consciousness	8 (27.6)	21 (72.4)	0.842
Runny nose	13 (48.1)	14 (51.9)	0.028
Arthralgia	3 (13.0)	20 (87.0)	0.083
Smell and taste distortion	9 (69.2)	4 (30.8)	0.003^c^
Urine issues	4 (44.4)	5 (55.6)	0.296^c^
Skin rash	3 (75.0)	1 (25.0)	0.077^c^

^a^Chi square test

^b^Mann-Whitney U tests

^c^Fisher exact test

A higher percentage of patients who had severe COVID-19 at admission were patients who were obese, had hypertension, diabetes, unknown immunodeficiencies, had not received the BCG vaccine, and had not received the first dose or second dose of the COVID-19 vaccine. A higher percentage of patients with fever (76.3%); cough or sore throat (74.7%); shortness of breath (82.5%); and severe dehydration (84.9%) were significantly associated with severe COVID-19 cases. In contrast, vomiting or nausea (57.7%); abdominal pain (58.0%); runny nose (51.9%); and smell and taste distortion (30.8%) were significantly associated with lower proportions of severe COVID-19 cases (Table 3).

Severe COVID-19 patients had significantly longer median durations from symptom onset to hospital admission (4 days), and higher medians of temperature (37.5 °C), heart rate (93 beats/min) and respiratory rate (20 breaths/min). In contrast, median oxygen saturation SpO2 (%) was significantly lower in severe COVID-19 patients compared to non-severe COVID-19 patients (95) (Table 4).

**Table 4:** Association between clinical measures on admission, and laboratory results with COVID-19 severity on admission

Variables	Non severe		Severe		P-value^a^
	Mean/median	± SD/IQR	Mean/Median	± SD/IQR	
Duration from symptoms to admission (days)	2	4	4	5	< 0.001
Clinical variables					
Systolic BP (mmHg)	125	24	129	26	0.090
Diastolic BP (mmHg)	78	16	77	15	0.560
Temperature (°C)	37.0	1.3	37.5	1.5	< 0.001
HR (Beats/Min)	89	24	93	26	0.010
RR (Breaths/Min)	18	4	20	7	< 0.001
SpO2 (%)	98	3	95	8	< 0.001
Glasgow coma score	15	0	15	0	0.070
Laboratory variables					
CBC					
WBC (× 10^9^/l)	6.10	4.10	6.80	4.08	0.002
Hb (g/dL)	13.05	2.63	13.10	2.40	0.806
Platelets (× 10^9^/l)	208.00	107.00	209.00	117.00	0.710
ANC (10^3/ul)	4.27	3.14	5.10	4.02	< 0.001
ALC (10^3/ul)	1.13	0.93	0.92	0. 59	0.001
Coagulation profile					
PT (Secs)	12.70	2.17	12.20	2.30	0.080
aPTT (Secs)	36.40	± 5.80	36.20	± 6.10	0.940^b^
INR	1.03	0.16	1.04	0.15	0.890
D-dimer (mg/L)	0.61	0.65	0.75	0.71	0.050
Electrolytes					
Sodium (mEq/L)	136.00	5.00	135.00	6.00	0.040
Potassium (mEq/L)	4.00	0.77	3.90	0.79	0.810
Renal function					
Creatinine (umol/L)	73.50	35.05	87.50	35.90	< 0.001
Urea (mmol/L)	5.71	5.00	5.89	4.20	0.830
Liver function					
Albumin (g/l)	35.00	12.00	31.00	9.48	< 0.001
Bilirubin, total (umol/l)	7.10	6.25	9.30	7.60	0.002
ALT/SGPT (U/L)	33.00	36.50	37.50	37.00	0.012
AST/SGOT (U/L)	35.00	33.00	41.00	34.00	0.040
Triglycerides (mmol/L)	2.09	± 0.54	1.70	± 0.69	0.163^b^
GGT (U/L)	39.00	72.00	32.00	38.00	0.076
Inflammatory markers					
ESR (mm/1 h)	40.00	28.00	59.80	29.60	0.020
Ferritin (ng/mL)	280.00	498.00	400.00	598.00	0.140
Procalcitonin (ng/mL)	0.10	0.25	0.15	0.27	0.001
Lactate (mmol/L)	1.30	0.54	1.50	0.92	0.210
LDH (U/L)	281.50	230.00	389.00	229.00	< 0.001
CRP (mg/l)	37.40	83.30	83.00	106.60	< 0.001
IL_6 (pg/mL)	45.00	74.50	21.20	88.90	0.574
Cardiac injury indicators					
Troponin (ng/mL)	0.008	0.021	0.008	0.170	0.770
CK (U/L)	130.000	202.000	115.500	180.500	0.720

^a^Mann-Whitney U tests

^b^Independent samples t-test

Notable differences in laboratory results were observed between severe and non-severe cases. Severe cases exhibited higher medians for white blood count (WBC) (6.8 × 10^9/L), absolute neutrophil count (ANC) (5.10 × 10^3/ul), and lower median absolute lymphocyte (ALC) (0.9 × 10^3/ µL). Furthermore, electrolyte and renal function markers in the severe group demonstrated differences, with a lower median sodium concentration (135 mEq/L) and a higher median creatinine level (87.5 µmol/L). Severe COVID-19 patients exhibited significantly higher medians of total bilirubin (9.3umol/l); alanine transaminase (ALT/SGPT) (37.5 U/L); and aspartate transaminase (AST/SGOT) (41 U/L) compared to non-severe patients. On the other hand, the median albumin level was lower in severe COVID-19 patients (31 g/l). Inflammatory markers such as erythrocyte sedimentation rate (ESR) (59.8 mm/1 h), lactate dehydrogenase (LDH) (389 U/L), procalcitonin (0.15 ng/mL), and C-reactive protein (CRP) (83 mg/l) were significantly higher in the severe COVID-19 group compared to the non-severe group (Table 4).

### Multiple Logistic Regression

Table 5 presents results of the multivariable logistic regression analysis. Model 4, which included all significant study variables, provided the best fit to the data with a Pseudo R^2^ value of 0.738 based on McFadden estimates compared to 0.143 for Model 1 (sociodemographic characteristics, smoking history, and past medical history), 0.334 for Model 2 (symptoms, and clinical measures), and 0.156 for Model 3 (laboratory results).

**Table 5:** Predictors for COVID-19 severity using multivariable logistic regression

	aOR	CI (95%)	p-value
Model 1: Sociodemographic characteristics, smoking history, and past medical history (N = 400)			
BMI: Normal	1		
Overweight	1.371	0.778,2.416	0.275
Obese	2.383	1.332,4.263	0.003
Area of residence (Dubai vs Sharjah/Ajman)	2.120	1.346,3.339	0.001
Crowding (1–2 person/room vs 3 or more person/room)	1.919	1.144,3.221	0.014
Smoking status (Non-smoker/former vs Current smoker)	0.367	0.182,0.740	0.005
COVID-19 first dose vaccination (no vs yes)	0.595	0.377,0.939	0.026
* Pseudo R*^*2*^ = *0.143*			
Model 2: Symptoms, and clinical measures (N = 673)			
Severe dehydration (no vs yes)	3.219	2.161,4.795	< 0.001
Fever (no vs yes)	1.990	1.272,3.113	0.003
Cough or sore throat (no vs yes)	1.607	1.032,2.502	0.036
Shortness of breath (no vs yes)	1.921	1.294,2.853	0.001
Oxygen saturation	0.853	0.801,0.907	< 0.001
Duration from symptoms to admission	1.055	1.006,1.105	0.026
* Pseudo R*^*2*^ = *0.334*			
Model 3: Laboratory results (N = 309)			
ANC	1.263	1.121,1.424	< 0.001
ALC	0.540	0.323,0.905	0.019
Bilirubin	1.036	1.004,1.069	0.026
* Pseudo R*^*2*^ = *0.156*			
Model 4: Sociodemographic characteristics, smoking history, past medical history, symptoms, clinical measures, and laboratory results (N = 127)			
BMI: Normal	1		
Overweight	1.678	0.376,7.490	0.479
Obese	14.040	2.204,89.450	0.005
Diabetes (no vs yes)	11.140	2.653,46,797	< 0.001
Severe dehydration (no vs yes)	6.722	1.678,26.931	0.007
Cough or sore throat (no vs yes)	5.355	1.241,23.117	0.025
AST/SGOT	1.055	1.016,1.095	0.005
* Pseudo R*^*2*^ = 0.738			

*aOR* adjusted odds ratio

Model 1: Includes 13 variables as follows: gender, marital status, age, income, BMI, area of residence, crowding, smoking status, hypertension, diabetes, BCG, COVID-19 first dose vaccination, and COVID-19 s dose vaccination

Model 2: Includes 12 variables as follows: Duration from symptoms to admission, severe dehydration, fever, cough or sore throat, shortness of breath, abdominal pain, vomiting, runny nose, temperature, heart rate, respiratory rate, and oxygen saturation

Model 3: Includes 11 variables as follows: WBC, ANC, ALC, Sodium, Creatinine, Albumin, Bilirubin, ALT/SGPT, AST/SGOT, Procalcitonin, and CRP

Model 4: Includes 35 variables as follows: gender, marital status, age, income, BMI, area of residence, crowding, smoking status, hypertension, diabetes, BCG, COVID-19 first dose vaccination, COVID-19 s dose vaccination, severe dehydration, fever, cough or sore throat, shortness of breath, abdominal pain, vomiting, runny nose, temperature, heart rate, respiratory rate, oxygen saturation, WBC, ANC, ALC, Sodium, Creatinine, Albumin, Bilirubin, ALT/SGPT, AST/SGOT, Procalcitonin, and CR

In Model 1, predictors of worse COVID-19 severity were overweight (aOR 1.371; 95% CI: 0.778, 2.416), obesity (aOR 2.383; 95% CI 1.332, 4.263), area of residence (aOR 2.120; 95% CI 1.332, 4.263), and crowding (aOR 1.919; 95% CI 1.144, 3.221). However, protective factors of worse COVID-19 were smoking status (aOR 0.367; 95% CI 0.182, 0.740) and receiving the first dose of COVID-19 vaccination (aOR 0.595; 95%CI 0.377, 0.939).

Model 2 showed that severe dehydration (aOR 3.219; 95%CI 2.161, 4.795), cough or sore throat (aOR 1.607; 95%CI 1.032, 2.502), shortness of breath (aOR 1.921; 95%CI 1.294, 2.853) and the number of days from symptom onset to admission were associated with increased likelihood of worse COVID-19 (aOR 1.055; 95%CI 1.006, 1.105). On the other hand, for every one unit increase in oxygen saturation, the risk of worse COVID-19 severity decreased by 14.7% (aOR 0.853; 95%CI 0.801, 0.907).

In model 3, ANC and bilirubin levels were positively associated with COVID-19 severity. For every one-unit increase in ANC and bilirubin, the likelihood of worse COVID-19 severity increased by 26.3% (aOR 1.263; 95%CI 1.121, 1.424) and 3.6% (aOR 1.036; 95%CI 1.004, 1.069), respectively. On the other hand, for every one-unit increase in ALC, the odds of worse COVID-19 severity decreased by 46.0% (aOR 0.540; 95%CI 0.323, 0.905).

Model 4 revealed that diabetic patients were approximately 11 times more likely to have severe COVID-19 on admission (aOR 11.14; 95% CI 2.653–46, 797). Additionally, an increase in AST/SGOT was associated with a higher  likelihood of severe COVID-19 (aOR 1.055, 95% CI 1.016, 1.095).

## Discussion

While the WHO declares the end of the COVID pandemic, there remains a critical need to augment the body of evidence for future pandemic management strategies and emphasize the significance of ongoing comprehensive studies. This study provides insights into epidemiological characteristics, clinical aspects, laboratory features, and risk factors of COVID-19 patients hospitalized in the UAE.

Most patients in our study were classified as moderate to severe COVID-19 cases, with only about a third experiencing mild symptoms. This is consistent with a previous study in Abu Dhabi, UAE [17]. The lower percentage of mild hospitalized patients suggests that those with mild symptoms tend to manage their illness through self-care while hospitalization becomes necessary when symptoms worsen. Additionally, the relatively low occurrence of critical cases suggests it would be appropriate to group moderate, severe, and critical cases together as a single category, distinguishing them from patients’ mild symptoms.

Interestingly, our findings revealed that overcrowding was a risk factor for COVID-19 severity. This observation is consistent with a previous study from Canada that linked both the spread and severity of COVID-19 to crowded conditions [18]. Despite the UAE’s reputation as a financial centre with modern infrastructure, the country faces unique challenges due to its substantial population of migrants, including construction laborers, and domestic workers living in shared accommodations. Such living arrangements create an environment conducive to the rapid transmission of infectious diseases [7]. Implementing regulations that specify minimum space per person and ensure adequate ventilation in shared accommodations can play a crucial role in preventing infectious disease transmission and safeguarding the well-being of individuals.

Another interesting finding in our study was the potential protective effect of smoking against severe COVID-19. Traditionally, tobacco products have been associated with negative effects on lung function in individuals with infectious diseases [9]. However, in the case of COVID-19, studies have reported a lower risk of severe COVID-19 outcomes among smokers [10]. One possible mechanism proposed to explain this effect involves nicotine’s potential to reduce the release of pro-inflammatory cytokine [19]. Another probable explanation for the protective effect of smoking against severe COVID-19 in our study population could be related to selection factors. It is possible that smokers in our study were more hospitalized than non-smokers, even in cases when their symptoms were mild, creating an impression that smoking was protective. Nevertheless, the association with COVID-19 is intricate and warrants further investigation.

Consistent with previous findings, our study demonstrated that receiving the first dose of a COVID-19 vaccine provided protection against severe COVID-19. The initial vaccine dose triggers the immune system to produce antibodies and memory cells capable of combating the virus. [20]. While the development of the immune response may vary among individuals and takes time, most COVID-19 vaccines have proven highly effective in reducing hospitalization rates, disease severity, and mortality with just a single dose [21]. Even though our study included participants before the availability of COVID-19 vaccination, the protective impact of the UAE’s nationwide vaccination program was evident. The UAE achieved nationwide immunity by providing free COVID-19 vaccines to all residents and citizens, becoming the first nation to successfully vaccinate 100% of eligible population groups [22].

Our study also found that diabetes and obesity were associated with worse COVID-19 outcomes consistent with previous research [8]. Given the high prevalence of diabetes and obesity in the UAE, understanding the complicated and multidirectional relationship between these conditions and COVID-19 is crucial [23]. Both conditions are recognized as part of the same interconnected pathophysiological mechanism that influences COVID-19 outcomes. Obesity and diabetes can lead to chronic inflammation, compromised immune responses, and alterations in pulmonary function, all contributing to more severe disease outcomes [8, 24].

Regarding symptoms, our results revealed a significant association between COVID-19 severity and the duration of symptoms from onset to hospital admission, aligning with a European study [25]. This alignment suggests that the longer the duration of symptoms before hospitalization, the higher the likelihood of experiencing severe COVID-19 outcomes. Among our severe patients, the most common symptoms included fever, cough, shortness of breath, severe dehydration, and low oxygen saturation levels, indicating an association with acute respiratory distress syndrome (ARDS). ARDS is characterized by rapid breathing, shortness of breath, and reduced oxygen saturation. Dehydration in severe COVID-19 patients can result from viral infection of the gastrointestinal tract or reduced fluid intake due to loss of appetite, nausea, vomiting, or difficulty swallowing [26].

Severe COVID-19 patients in our study had elevated ANC levels and decreased ALC levels. These findings align with a previous meta-analysis, which demonstrated that elevated neutrophil count and decreased lymphocyte count at admission can predict COVID-19 severity [27]. It’s important to note that hospitalized patients may display abnormal laboratory results due to various factors such as systemic inflammation response syndrome, drug-induced liver injury, or multiorgan failure. Therefore, relying solely on these markers as a single biomarker predictor should be approached with caution [6]. One plausible explanation for these findings is that lymphocytes play a key role in regulation of the inflammatory response, and their loss due to persistent infection may lead to immune system suppression and the nonresolution of inflammation. Consequently, the medulla in the brain may produce more neutrophils to combat the invading pathogen [28].

The present study also revealed elevated levels of AST and total bilirubin in severe COVID-19 patients, indicating altercations in liver biochemistry. This association between severe COVID-19 infection and liver involvement has been reported in a systematic literature review conducted by Afra et al. [29]. AST levels typically indicate hepatocyte (liver cell) injury, while elevated bilirubin levels suggest bile duct damage. These markers serve as indicators for assessing the severity of liver involvement in COVID-19 patients and guiding appropriate management strategies. Several factors contribute to the progression of liver injury in COVID-19, including direct viral invasion of liver cells, cytokine storms that trigger liver inflammation and damage, and ischemia resulting from impaired blood flow to the liver [29, 30].

## Strengths and Limitations

To the best of our knowledge, this study represents the first comprehensive examination of clinical and epidemiological characteristics of COVID-19 hospitalized patients in the UAE, encompassing multiple centres and spanning various phases of the pandemic. The study provides insights on potential risk factors associated with the severity of COVID-19, which can aid decision makers and inform population level public health strategies. Although prior infections and vaccinations have significantly reduced the overall risk of severe outcomes compared to the early stages of the pandemic, the risk factors identified in this study are likely to continue influencing relative risks in the future.

However, our study does have limitations. The clinical homogeneity of our study participants was compromised due to the 21-month period during which the clinical landscape of COVID-19 naturally evolved. The emergence of different variants of the virus across waves of the pandemic may have contributed to evolving clinical presentations of the disease over time. We were unable to consider the strains in our analysis since the data in the medical records of the four participating hospitals lacked this information. Furthermore, our study had low response rate, which was mostly attributed to the fact that many of the participants were non reachable expatriates who had left the country or changed their phone numbers. In addition, incomplete laboratory results exist as not all patients underwent the same battery of tests across participating hospitals. The absence of uniform data across all cases may affect the comprehensiveness of our analysis, potentially missing critical information that could further elucidate COVID-19 characteristics and risk factors. Moreover, our study may be susceptible to survival bias as it excluded individuals who passed away during the follow-up period. This exclusion may skew our findings towards less severe cases and fail to capture valuable insights into the full spectrum of COVID-19 severity.

However, while we acknowledge the constraints and potential biases inherent in our research, these limitations do not diminish the importance and knowledge gained from this study. Our findings offer insights that can significantly benefit our understanding and management of COVID-19.

## Conclusion

Our findings highlight the association between crowding, obesity, diabetes, severe dehydration, cough or sore throat, shortness of breath, increased days from symptoms onset to admission, elevated ANC levels, and elevated AST/SGOT levels with an increased risk of severe COVID-19 symptoms. This underscores the importance of rigorous screening protocols in healthcare facilities, enabling early identification and intervention for individuals with these risk factors. Incorporating risk factors into surveillance protocols will help healthcare systems identify high risk individuals, allocate resources efficiently and respond rapidly to outbreaks ultimately improving public health outcomes.

### Supplementary Information

Below is the link to the electronic supplementary material.

Supplementary file1 (DOCX 88 KB)

Supplementary file2 (DOCX 87 KB)

## Data Availability

The data that support the findings of this study are not openly available due to reasons of confidentiality and are available from the corresponding author upon reasonable request.

## Abbreviations

COVID-19: Coronavirus disease of 2019
UAE: United Arab Emirates
aOR: Adjusted odds ratio
CI: Confidence intervals
AST/SGOT: Aspartate aminotransferase/serum glutamic-oxaloacetic transaminase
ALC: Absolute lymphocyte count
ANC: Absolute neutrophile count
WHO: World Health Organization
RT-PCR: Real time Reverse Transcriptase Polymerase Chain Reaction
ISARIC: International Severe Acute Respiratory and Emerging Infection Consortium
BMI: Body Mass Index
PaO2: Partial pressure of arterial oxygen
FiO2: Fraction of inspired oxygen
ACE: Angiotensin-converting enzyme inhibitors
ARBS: Angiotensin II receptor blockers
NSAID: Non-steroidal anti-inflammatory drug
BCG: Bacillus calmette-guérin
GGT: Gamma-glutamyl transferase
aPTT: Activated partial thromboplastin time
ESR: Erythrocyte sedimentation rate
CK: Creatinine kinase
IgG: Immunoglobin G
LDH: Lactate dehydrogenase
CRP: C-reactive protein
ESR: Erythrocyte sedimentation rate
INR: International normalized ratio
CBC: Complete blood count
WBC: White blood count
Hb: Hemoglobin
PT: Prothrombin time
GGT: Gamma-glutamyl transferase
LDH: Lactate dehydrogenase
CRP: C-reactive protein
IQR: Interquartile range
BP: Blood pressure

## References

[1] WHO_a. World Health Organization (WHO). Statement on the fifteenth meeting of the IHR Emergency Committee on the COVID-19 pandemic. 2023 Accessed on 31/03/2023]; Available from: https://www.who.int/news/item/05-05-2023-statement-on-the-fifteenth-meeting-of-the-international-health-regulations- (2005)-emergency-committee-regarding-the-coronavirus-disease- (covid-19)-pandemic.

[2] Wordlometers. COVID-19 Coronavirus Pandemic. Reported Cases and Deaths by Country or Territory. 2023 Accessed on 20–06–2023]; Available from: https://www.worldometers.info/coronavirus/

[3] Docherty AB (2020). Features of 20 133 UK patients in hospital with covid-19 using the ISARIC WHO Clinical Characterisation Protocol: prospective observational cohort study. BMJ.

[4] Huang C (2020). Clinical features of patients infected with 2019 novel coronavirus in Wuhan. China Lancet.

[5] Livingston E, Bucher K (2020). Coronavirus disease 2019 (COVID-19) in Italy. JAMA.

[6] Tan JY (2021). A comparative study on the clinical features of COVID-19 with non-SARS-CoV-2 respiratory viral infections. J Med Virol.

[7] GMI. United Arab Emirates Ppopulation Statistics. 2023 Accessed on 20–06–2023]; Available from: https://www.globalmediainsight.com/blog/uae-population-statistics/#:~:text=Majority%20of%20the%20UAE%20population,for%20Dubai%20is%2033.5%20years.

[8] Al Bastaki NA (2022). An evaluation of non-communicable diseases and risk factors associated with COVID-19 disease severity in Dubai, United Arab Emirates: an observational retrospective study. Int J Environ Res Public Health.

[9] He Y (2022). Association between smoking and COVID-19 severity: a multicentre retrospective observational study. Medicine (Baltimore).

[10] Simons D (2021). The association of smoking status with SARS-CoV-2 infection, hospitalization and mortality from COVID-19: a living rapid evidence review with Bayesian meta-analyses (version 7). Addiction.

[11] Irizar P (2023). Ethnic inequalities in COVID-19 infection, hospitalisation, intensive care admission, and death: a global systematic review and meta-analysis of over 200 million study participants. EClinicalMedicine.

[12] Al Mansoori L (2021). Epidemiological characteristics of children with coronavirus at a joint commission-accredited hospital in the United Arab Emirates. J Family Med Prim Care.

[13] Hachim IY (2021). The inflammatory biomarkers profile of hospitalized patients with COVID-19 and its association with patient's outcome: a single centered study. PLoS ONE.

[14] WHO_b, A Healthy Lifestyle-WHO Recommendations. 2010. Available online: https://www.who.int/europe/news-room/fact-sheets/item/a-healthy-lifestyle---who-recommendations#:~:text=To%20ensure%20a%20healthy%20lifestyle,promote%20and%20support%20healthy%20lifestyles. Aaccessed on 20–06–2023.

[15] Wu Y, et al. Evolution and major changes of the diagnosis and treatment protocol for COVID-19 patients in China 2020–2023. Health Care Sci. 2023;2:135–152. 10.1002/hcs2.45 10.1002/hcs2.45 PMC11080729 38939112

[16] IBM Crop. Released 2021. IBM SPSS Statistics for Windows, Version 28.0. Armonk, NY: IBM Corp.

[17] Hosani FA (2021). Epidemiology of asymptomatic and symptomatic Coronavirus Disease 2019 confirmed cases in the Emirate of Abu Dhabi, United Arab Emirates: observational study. Medicine (Baltimore).

[18] Brown KA (2021). Association between nursing home crowding and COVID-19 infection and mortality in Ontario. Canada JAMA Intern Med.

[19] Pavlov VA, Tracey KJ (2005). The cholinergic anti-inflammatory pathway. Brain Behav Immun.

[20] Barnes E (2023). SARS-CoV-2-specific immune responses and clinical outcomes after COVID-19 vaccination in patients with immune-suppressive disease. Nat Med.

[21] Rotshild V (2021). Comparing the clinical efficacy of COVID-19 vaccines: a systematic review and network meta-analysis. Sci Rep.

[22] UAE portal, 2022. Vaccines against COVID-19 in the UAE. Accessed on 22–06–2023. ]; Available from: https://u.ae/en/information-and-services/justice-safety-and-the-law/handling-the-covid-19-outbreak/vaccines-against-covid-19-in-the-uae.

[23] Al Sabbah H (2023). Nutrition situation analysis in the UAE: a review study. Nutrients.

[24] Kruglikov IL, Shah M, Scherer PE (2020). Obesity and diabetes as comorbidities for COVID-19: Underlying mechanisms and the role of viral-bacterial interactions. Elife.

[25] Palmieri L (2020). Clinical characteristics of hospitalized individuals dying with COVID-19 by age group in Italy. J Gerontol A Biol Sci Med Sci.

[26] Pan L (2020). Clinical characteristics of COVID-19 patients with digestive symptoms in Hubei, China: a descriptive, cross-sectional. Multicenter Study Am J Gastroenterol.

[27] Henry B (2020). Lymphopenia and neutrophilia at admission predicts severity and mortality in patients with COVID-19: a meta-analysis. Acta Biomed.

[28] Liu X (2016). Prognostic significance of neutrophil-to-lymphocyte ratio in patients with sepsis: a prospective observational study. Mediators Inflamm.

[29] Shokri Afra H (2020). Positive association between severity of COVID-19 infection and liver damage: a systematic review and meta-analysis. Gastroenterol Hepatol Bed Bench.

[30] Robinson MW, Harmon C, O'Farrelly C (2016). Liver immunology and its role in inflammation and homeostasis. Cell Mol Immunol.

