# Supplementary material for: Epidemiological and Clinical Characteristics Associated with COVID-19 Severity Among Hospitalized Patients in the United Arab Emirates: A Retrospective Multicentre Study
Source: J Epidemiol Glob Health. 2024 Feb 26;14(2):349–62. doi: 10.1007/s44197-024-00206-8 (PMC11176126; doi:10.1007/s44197-024-00206-8)
Supplement: Supplementary file 1 — Supplementary file1 (DOCX 88 KB) [file 44197_2024_206_MOESM1_ESM.docx]

**Supplemental files (Appendix 1):**  Association between sociodemographic characteristics, smoking history, past medical history, and symptoms on admission with COVID-19 severity on admission

| **Variables** | **Non severe (%)** | **Severe (%)** | **P-value** | **Variables** | **Non severe (%)** | **Severe (%)** | **P-value** |
| --- | --- | --- | --- | --- | --- | --- | --- |
| **Gender** |  |  |  | **Crowding** |  |  |  |
| Female | 35 | 65 | 0.009 | 1-2 person/room | 34.9 | 65.1 | 0.048 |
| Male | 25.9 | 74.1 |  | 3 or more person/room | 26.3 | 73.7 |  |
| **Marital status** |  |  |  | **Smoking status** |  |  |  |
| Single | 47.3 | 52.7 | 0.024 | Non-smoker/former smoker | 31.1 | 68.9 | 0.021 |
| Married | 32 | 68 |  | Current smoker | 47.1 | 52.9 |  |
| Divorced/widowed | 24.2 | 75.8 |  | **Smoking frequency** |  |  |  |
| **Age in years** |  |  |  | Not regular (not every day) | 34.7 | 65.3 | 0.527 |
| 18-29 | 58.1 | 41.9 | <0.001 | Regular (all 30 days) | 40.2 | 59.8 |  |
| 30-60 | 27.9 | 72.1 |  | **Smoking frequency** |  |  |  |
| 60+ | 25.8 | 74.2 |  | 1-5 | 35.8 | 64.2 | 0.577 |
| **Education level** |  |  |  | 6 and more | 41.2 | 58.8 |  |
| ≤ High school | 32.1 | 67.9 | 0.234 | **Anyone in the family smokes** | 38.9 | 61.1 | 0.102 |
| Bachelors | 30.6 | 69.4 |  | **Past surgery** | 34 | 66 | 0.096 |
| Postgraduate | 45.5 | 54.5 |  | **BMI** |  |  |  |
| **Ethnicity (570)** |  |  |  | Underweight | 80 | 20 | <0.001 |
| Asian | 30.5 | 69.5 | 0.727 | Normal | 36.4 | 63.6 |  |
| Arab | 33.6 | 66.4 |  | Overweight | 30.7 | 69.3 |  |
| Others | 30.8 | 69.2 |  | Obese | 22.4 | 77.6 |  |
| **Income/month AED** |  |  |  | **Comorbidities** |  |  |  |
| < 15000 | 28 | 72 | 0.034 | **Chronic cardiac disease** | 27.2 | 72.8 | 0.602 |
| 15000-29999 | 41.6 | 58.4 |  | **Hypertension** | 23.6 | 76.4 | 0.009 |
| ≥ 30000 | 30.1 | 69.9 |  | **Chronic pulmonary disease/Asthma** | 26.2 | 73.8 | 0.655 |
| **Work status** |  |  |  | **Chronic kidney disease** | 32.6 | 67.4 | 0.603 |
| Not working | 32.6 | 67.4 | 0.804 | **Chronic liver disease⁑** | 60 | 40 | 0.072 |
| Working | 31.6 | 68.4 |  | **Chronic neurological disease⁑** | 23.5 | 76.5 | 0.789 |
| **Occupation** |  |  |  | **Diabetes** | 24.5 | 75.5 | 0.031 |
| Labour/cleaner/delivery | 28.9 | 71.1 | 0.62 | **Malignant neoplasm** | 37.5 | 62.5 | 0.365 |
| Admin work | 32.1 | 67.9 |  | **Unknown immunodeficiencies** | 8.3 | 91.7 | 0.022 |
| Managerial | 30.6 | 69.4 |  | **Anaemia** | 38.1 | 61.9 | 0.365 |
| Business | 29.7 | 70.3 |  | **High cholesterol levels** | 28.7 | 71.3 | 0.92 |
| Health care worker | 45.8 | 54.2 |  | **Thyroid disease** | 26.5 | 73.5 | 0.717 |
| **Family history of consanguinity** | 33.1 | 66.9 | 0.805 | **Pre-existing medications** |  |  |  |
| **Area of residence** |  |  |  | **ACE inhibitors** | 27.1 | 72.9 | 0.709 |
| Dubai | 46 | 54 | <0.001 | **ARBS** | 26.4 | 73.6 | 0.639 |
| Sharjah/Ajman | 25.6 | 74.4 |  | **NSAID** | 24.5 | 75.5 | 0.434 |
| Other emirates | 22.2 | 77.8 |  | **Pressor Support ‡** | 30 | 70 | 0.957 |
| ‡ Fisher exact test | | | | | | | |
