# Supplementary material for: Epidemiological and Clinical Characteristics Associated with COVID-19 Severity Among Hospitalized Patients in the United Arab Emirates: A Retrospective Multicentre Study
Source: J Epidemiol Glob Health. 2024 Feb 26;14(2):349–62. doi: 10.1007/s44197-024-00206-8 (PMC11176126; doi:10.1007/s44197-024-00206-8)
Supplement: Supplementary file 2 — Supplementary file2 (DOCX 87 KB) [file 44197_2024_206_MOESM2_ESM.docx]

| **Supplemental files (Appendix 2):** Independent t-test and Mann Whitney U test for association between clinical measures on admission, and laboratory results with COVID-19 severity on admission. | | | | | |
| --- | --- | --- | --- | --- | --- |
| **Variables** | **Non severe** | | **Sever** | | **P-value** |
|  | **Mean*/Median** | **±SD/IQR** | **Mean*/Median** | **±SD/IQR** |  |
| **Duration from symptoms to admission (days)** | 2 | 4 | 4 | 5 | <0.001 |
| **Clinical variables** | | | | | |
| **Systolic BP (mmHg)** | 125 | 24 | 129 | 26 | 0.090 |
| **Diastolic BP (mmHg)** | 78 | 16 | 77 | 15 | 0.560 |
| **Temperature (**°**C)** | 37.0 | 1.3 | 37.5 | 1.5 | <0.001 |
| **HR (Beats/Min)** | 89 | 24 | 93 | 26 | 0.010 |
| **RR (Breaths/Min)** | 18 | 4 | 20 | 7 | <0.001 |
| **SpO2 (%)** | 98 | 3 | 95 | 8 | <0.001 |
| **Glasgow coma score** | 15 | 0 | 15 | 0 | 0.070 |
| **Lab variable** | | | | | |
| **CBC** | | | | | |
| WBC (×10^9^/l) | 6.10 | 4.10 | 6.80 | 4.08 | 0.002 |
| Hb (g/dL) | 13.05 | 2.63 | 13.10 | 2.40 | 0.806 |
| Platelets (×10^9^/l) | 208.00 | 107.00 | 209.00 | 117.00 | 0.710 |
| ANC (10^3/ul) | 4.27 | 3.14 | 5.10 | 4.02 | <0.001 |
| ALC (10^3/ul) | 1.13 | 0.93 | 0.92 | 0. 59 | 0.001 |
| **Coagulation profile** | | | | | |
| PT (Secs) | 12.70 | 2.17 | 12.20 | 2.30 | 0.080 |
| aPTT * (Secs) | 36.40 | ±5.80 | 36.20 | ±6.10 | 0.940 |
| INR | 1.03 | 0.16 | 1.04 | 0.15 | 0.890 |
| D-Dimer (mg/L) | 0.61 | 0.65 | 0.75 | 0.71 | 0.050 |
| **Electrolytes** | | | | | |
| Sodium (mEq/L) | 136.00 | 5.00 | 135.00 | 6.00 | 0.040 |
| Potassium (mEq/L) | 4.00 | 0.77 | 3.90 | 0.79 | 0.810 |
| **Renal function** | | | | | |
| Creatinine (umol/L) | 73.50 | 35.05 | 87.50 | 35.90 | <0.001 |
| Urea (mmol/L) | 5.71 | 5.00 | 5.89 | 4.20 | 0.830 |
| **Liver Function** | | | | | |
| Albumin (g/l) | 35.00 | 12.00 | 31.00 | 9.48 | <0.001 |
| Bilirubin, Total (umol/l) | 7.10 | 6.25 | 9.30 | 7.60 | 0.002 |
| ALT/SGPT (U/L) | 33.00 | 36.50 | 37.50 | 37.00 | 0.012 |
| AST/SGOT (U/L) | 35.00 | 33.00 | 41.00 | 34.00 | 0.040 |
| Triglycerides* (mmol/L) | 2.09 | ± 0.54 | 1.70 | ±0.69 | 0.163 |
| GGT (U/L) | 39.00 | 72.00 | 32.00 | 38.00 | 0.076 |
| **Inflammatory markers** | | | | | |
| ESR (mm/1hr) | 40.00 | 28.00 | 59.80 | 29.60 | 0.020 |
| Ferritin (ng/mL) | 280.00 | 498.00 | 400.00 | 598.00 | 0.140 |
| Procalcitonin (ng/mL) | 0.10 | 0.25 | 0.15 | 0.27 | 0.001 |
| Lactate (mmol/L) | 1.30 | 0.54 | 1.50 | 0.92 | 0.210 |
| LDH (U/L) | 281.50 | 230.00 | 389.00 | 229.00 | <0.001 |
| CRP (mg/l) | 37.40 | 83.30 | 83.00 | 106.60 | <0.001 |
| IL_6 (pg/mL) | 45.00 | 74.50 | 21.20 | 88.90 | 0.574 |
| **Cardiac injury indicators** | | | | | |
| Troponin (ng/mL) | 0.008 | 0.021 | 0.008 | 0.170 | 0.770 |
| CK (U/L) | 130.000 | 202.000 | 115.500 | 180.500 | 0.720 |
| * Independent t-test; data is summarized in mean and standard deviation | | | | | |
